# The VEGF- and PDGF-family of angiogenic markers have prognostic impact in soft tissue sarcomas arising in the extremities and trunk

**DOI:** 10.1186/1472-6890-14-5

**Published:** 2014-01-20

**Authors:** Thomas K Kilvaer, Eivind Smeland, Andrej Valkov, Sveinung W Sorbye, Roy M Bremnes, Lill-Tove Busund, Tom Donnem

**Affiliations:** 1 Department of Oncology, University Hospital of North Norway, Tromso, 9037 Norway; 2 Institute of Medical Biology, University of Tromso, Tromso, Norway; 3 Department of Clinical Pathology, University Hospital of North Norway, Tromso, Norway; 4 Institute of Clinical Medicine, University of Tromso, Tromso, Norway

**Keywords:** Angiogenesis, Sarcoma, Extremity, Trunk, FGF, PDGF, VEGF, Visceral, Retroperitoneal

## Abstract

**Background:**

Soft-tissue sarcomas are rare malignant tumors of mesenchymal lineage that can arise in any part of the body. Prognosis, and hence also treatment may vary according to histologic subtype and localization. Angiogenesis is the process of forming new blood vessels from pre-existing ones. The deregulation of this process is thought to be an important step in malignant transformation. This study investigates the prognostic impact of platelet derived growth factor- (PDGF), vascular endothelial growth factor- (VEGF) and fibroblast growth factor (FGF) families in soft-tissue sarcomas of the extremities & trunk (ET) and visceral & retroperitoneal (VR) locations.

**Methods:**

Tumor samples from 181 patients (115 ET and 66 VR) with resected soft tissue sarcomas were collected and tissue microarrays were constructed. Immunohistochemistry was used to evaluate angiogenic marker expression. Recurrence-free survival (RFS), metastasis-free survival (MFS) and disease-specific survival (DSS) were used as endpoints in prognostic impact assessment.

**Results:**

In univariate analyses, almost all investigated angiogenic markers had prognostic impact in the ET group. In contrast, only FGFR-1 showed any significant prognostic impact in the VR group. In the multivariate analyses, PDGF-D (HR = 1.863, 95% CI = 1.057-3.283, P = 0.031), VEGFR-1 (HR = 2.106, 95% CI = 1.038-4.272, P = 0.039) and VEGF-A (HR 2.095, 95% CI 1.028-4.271, P = 0.042) were independent negative prognosticators for DSS, MFS and RFS, respectively, in the ET group. FGFR-1 was an independent positive prognosticator for DSS (HR = 0.243, 95% CI = 0.095-0.618, P = 0.003) in the VR group.

**Conclusions:**

Angiogenic molecules from the PDGF and VEGF families have prognostic impact in soft-tissue sarcomas arising in the ET, but not in VR locations. In the latter histological grade and resection margins are the most important prognostic factors.

## Background

Soft tissue sarcomas (STS) constitute a highly heterogeneous collection of tumors comprising over 50 histological subtypes, arising from mesenchymal tissue and capable of forming tumors in all parts of the human body [1]. This group amounts to 0.5-1% of the annual tumor burden with a mortality of about 40-60%, resulting in an estimated 11 280 cases and 3 900 deaths in the US in 2012 [2]. It is good practice to distinguish between STSs arising in the extremity & trunk (ET), head & neck (HN) and visceral & retroperitoneal (VR) localizations as treatment and prognosis vary widely according to localization [3]. Further subdivision, according to histological type, malignancy grade, stage and vascular invasion among others, can be conducted [3]. Definitive treatment is radical surgery followed by radiotherapy in case of non-radical surgical margins [4]. Adjuvant chemotherapy for adult STS is still under investigation, and hence the routine use of such treatment is today limited to the palliative setting [5].

Angiogenesis is the process of forming new blood vessels from pre-existing ones. Folkman and coworkers proved this to be a pivotal step in carcinogenesis by showing that tumors would not grow beyond > 2 mm in diameter without forming vasculature [6,7]. In 2001, Hanahan and Weinberg, suggested angiogenesis as one of the hallmarks of cancer [8] and in the 2011 updated version angiogenesis was still considered one of the most important aspects of cancer progression [9].

Vascular endothelial growth factors (VEGF) and receptors (VEGFR) are pivotal in endothelial cell proliferation and sprouting during angio- and lymphangiogenesis [10]. Platelet-derived growth factors (PDGF) and receptors (PDGFR) play an important part in the regulation of tumor stroma through the recruitment of pericytes and vascular smooth muscle cells helping to stabilize newly formed vessels and through stimulation of stromal cells to produce VEGF-A and thus drive angiogenesis [11,12]. Fibroblast growth factors (FGF) and receptors (FGFR) drives endothelial cell proliferation and sprouting and activate several molecules involved in extracellular matrix remodelling including matrix metallo-proteinases and urokinase-like plasminogen activator [13].

Our group has previously reported on the expression of VEGF, PDGF and FGF families of growth factors in STSs of all sites [14-16]. This report investigates the differential impact of these growth factors in STSs arising in ET versus VR localizations.

## Methods

### Patients and clinical samples

Primary tumor tissue from anonymized patients diagnosed with STS at the University Hospital of North-Norway and the Hospitals of Arkhangelsk County, Russia, from 1973 through 2006, were collected. In total 496 patients were registered from the hospital databases. Of these, 388 patients were excluded from the study because of: missing clinical data (n = 86), inadequate formalin-fixed paraffin-embedded (FFPE) tissue blocks (n = 161), no surgery performed and/or metastasis present at the time of diagnosis (n = 55) or head and neck sarcomas (n =13). Thus 115 patients with STSs of the extremities and trunk wall and 66 patients with STSs of visceral or retroperitoneal origin, with complete medical records and FFPE tissue blocks were eligible.

This report includes follow-up data as of September 2009. The median follow-up was 53.9 (range 0.5-391.7) months for extremity and trunk patients and 59.4 (range 0.10-366.7) months for visceral and retroperitoneal patients. Complete demographic and clinical data were collected retrospectively. Formalin-fixed and paraffin-embedded tumor specimens were obtained from the archives of the Departments of Pathology at the University Hospital of North-Norway and the Hospitals of Arkhangelsk County, Russia. The tumors were graded according to the French Fédération Nationale des centres de Lutte Contre le Cancer (FNCLCC) system and histologically subtyped according to the World Health Organization guidelines [1,17]. Wide resection margins were defined as wide local resection with free microscopic margins or amputation of the affected limb or organ.

### Microarray construction

All sarcomas were histologically reviewed by two trained pathologists (S. Sorbye and A. Valkov) and the most representative areas of tumor cells (neoplastic mesenchymal cells) were carefully selected and marked on the hematoxylin and eosin (H/E) slide and sampled for the tissue microarray (TMA) blocks. The TMAs were assembled using a tissue-arraying instrument (Beecher Instruments, Silver Springs, MD). The Detailed methodology has been previously reported [18]. Briefly, we used a 0.6 mm diameter stylet, and the study specimens were routinely sampled with four replicate core samples from different areas of neoplastic tissue. Normal tissue from the patients was used as staining control.

To include all core samples, 12 TMA blocks were constructed. Multiple 5-μm sections were cut with a Micron microtome (HM355S) and stained by specific antibodies for immunohistochemistry (IHC) analysis.

### Immunohistochemistry

The applied antibodies were subjected to in-house validation by the manufacturer for IHC analysis on paraffin-embedded material. The detailed methodology has previously been reported [14-16].

### Scoring of immunohistochemistry

The ARIOL imaging system (Genetix, San Jose, CA) was used to scan the slides of antibody staining of the TMAs and the dominant staining intensity was scored as: 0 = negative; 1 = weak; 2 = intermediate; 3 = strong semi-qantitively on computer screen. The detailed methodology has previously been reported and cut-off values chosen were the same as in our previous studies [14-16]. High expression in tumor cells were defined as ≥ 1 (VEGF-C), ≥ 1.5 (PDGF-A, PDGF-C, PDGF-B, VEGF-A, VEGF-D, VEGFR-1-2 and -3) and ≥ 2 (PDGF-D, PDGFR-α, PDGFR-β, FGF2 and FGFR-1).

### Statistical methods

All statistical analyses were done using the statistical package SPSS (Chicago, IL), version 16. The IHC scores from each observer were compared for interobserver reliability by use of a two-way random effect model with absolute agreement definition. The intraclass correlation coefficient (reliability coefficient) was obtained from these results. The Chi-square test and Fishers Exact test were used to examine the association between molecular marker expression and various clinicopathological parameters. Univariate analyses were done using the Kaplan-Meier method, and statistical significance between survival curves was assessed by the log-rank test. Disease-specific survival (DSS) was determined from the date of diagnosis to the time of cancer related death. Metastasis-free survival (MFS) was defined from the date of diagnosis to the clinical appearance of the first metastasis. Recurrence-free survival (RFS), was defined from the date of diagnosis to the clinical appearance of the first recurrence. To assess the independent value of different pretreatment variables on survival, metastasis and local recurrence, in the presence of other variables, multivariate analyses were carried out using the Cox proportional hazards model. Only variables of significant value from the univariate analyses were entered into the Cox regression analysis. Probability for stepwise entry and removal was set at .05 and .10, respectively. The significance level used for all statistical tests was P < 0.05.

### Ethical clearance

The Norwegian National Data Inspection Board and The Regional Committee for Research Ethics (Northern Norway) approved the study.

## Results

### Clinicopathological variables

The clinicopathological variables are summarized in Table 1. In the ET group, comprising 115 patients, median age was 59 (range 0-89) years, 50% of the patients were male, 67 patients were Norwegian and 48 Russian and 68% of the tumors were located in the extremities. Of the histological subtypes represented, 48 were undifferentiated pleomorphic sarcomas, 18 liposarcomas, 12 fibrosarcomas, 10 synovial sarcomas, 9 leiomyosarcomas, 5 angiosarcomas, 5 rhabdomyosarcomas, 5 malignant peripheral nerve sheath tumors (MPNST) and 3 sarcoma not otherwise specified (NOS).

**Table 1:** Prognostic clinicopathological variables as predictors for disease-specific survival, metastasis and local recurrence in patients with resected Extremitiy & Trunk and Visceral & Retroperitoneal soft-tissue sarcomas (univariate analyses, log rank test, n = 115 and 66 respectively)

	**Extremity & trunk**							**Visceral & retroperitoneal**						
		**Disease-specific survival**		**Metastasis-free survival**		**Recurrence-free survival**			**Disease-specific survival**		**Metastasis-free survival**		**Recurrence-free survival**	
**Characteristics**	**Patients (n)**	**5-Year survival (%)**	**P**	**5-Year survival (%)**	**P**	**5-Year survival (%)**	**P**	**Patients (n)**	**5-Year survival (%)**	**P**	**5-Year survival (%)**	**P**	**5-Year survival (%)**	**P**
**Age**														
≤ 20 years	12	42	0.431	42	0.129	73	0.690	1	0	<0.001	100	0.112	100	0.786
21-60 years	48	61		63		69		36	71		67		71	
> 60 years	55	55		69		64		29	43		44		62	
**Gender**														
Male	57	56	0.298	66	0.368	66	0.759	15	79	0.039	86	0.022	66	0.799
Female	58	54		61		67		51	51		48		68	
**Patient nationality**														
Norwegian	67	65	0.004	74	0.008	71	0.249	54	62	0.051	59	0.122	66	0.892
Russian	48	42		48		57		12	36		46		59	
**Histological entity**														
Pleomorphic sarcoma	48	42	0.004	61	0.001	56	0.664	6	50	0.917	67	0.264	40	0.274
Leiomyosarcoma	9	100		78		78		39	55		46		77	
Liposarcoma	18	83		94		89		13	62		81		54	
Fibrosarcoma	12	57		67		55		0						
Angiosarcoma	5	40		20		67		2	50		50		50	
Rhabdomyosarcoma	5	60		60		60		1			100		100	
MPNST	5	53		60		60		4	67		100		100	
Synovial sarcoma	10	13		30		64		1	100		0		0	
Sarcoma NOS	3	100		67		67		0						
**Tumor size**														
< 5 cm	38	70	0.048	81	0.053	74	0.085	11	82	0.107	73	0.259	89	0.006
5-10 cm	45	48		53		61		24	62		62		70	
> 10 cm	30	49		58		62		31	45		45		49	
Missing	2													
**Malignancy grade**														
1	29	89	<0.001	89	0.001	85	0.054	23	78	0.005	77	0.051	86	0.046
2	41	56		65		60		29	46		42		58	
3	45	35		43		57		14	46		47		49	
**Vascular invasion**														
Absent	64	70	<0.001	85	<0.001	79	<0.001	43	59	0.656	60	0.847	66	0.675
Present	50	35		33		43		20	51		54		78	
Missing	1							3						
**Tumor depth**														
Superficial	12	91	0.010	100	0.012	91	0.041	32						
Deep	103	51		59		63		34						
**Resection margins**														
Wide	61	66	0.004	72	0.045	82	<0.001	50	65	0.021	59	0.654	90	<0.001
Non-wide	54	44		54		46		16	50		53		44	

*Abbreviations*: *MPNST* Malingnant peripheral nerve sheat tumor, *NOS* Not otherwise specified.

In the VR group, median age was 58 (range 13-88) years, 23% of the patients were male and 54 patients were Norwegian and 12 Russian. Of the histological subtypes represented, 39 were leiomyosarcomas, 13 liposarcomas, 6 pleomorphic sarcomas, 4 neurofibrosarcomas/MPNSTs, 2 angiosarcomas, 1 rhabdomyosarcoma and 1 synovial sarcoma.

### Interobserver variability

Interobserver scoring agreement was tested for PDGF-B, PDGFR-α, VEGF-C, VEGFR-3, FGF2 and FGFR1 and found to be good (0.77-0.90, P < 0.001) [14-16].

### Univariate analyses

The impact of the clinicopathological variables on DSS, MFS and RFS in the ET group are summarized in Table 1. Patient nationality (P = 0.004), histological entity (p = 0.004), tumor size (p = 0.048), malignancy grade (P < 0.001), vascular invasion (P <0.001), tumor depth (P = 0.010) and resection margins (P = 0.004) were all prognostic indicators of DSS. Patient nationality (P = 0.008), histological entity (P = 0.001), malignancy grade (P = 0.001), vascular invasion (P < 0.001), tumor depth (P = 0.012) and resection margins (P = 0.045) were prognostic indicators of MFS. Finally, vascular invasion (P < 0.001), tumor depth (P = 0.041) and resection margins (P < 0.001) were prognostic indicators of RFS.

The impact of the angiogenic markers on DSS, MFS and RFS in the ET group are summarized in Table 2. PDGF-A (P = 0.035), PDGF-B (P = 0.006), PDGF-C (P = 0.032), PDGF-D (P = 0.003), PDGFR-α (P = 0.002), PDGFR-β (P = 0.029), VEGF-A (P = 0.001), VEGFR-1 (P = 0.001) and FGF2 (P = 0.033) were prognostic indicators of DSS. PDGF-A (P = 0.007), PDGF-B (P = 0.003), PDGFR-α (P = 0.002), PDGFR-β (P = 0.002), VEGF-A (P = 0.001), VEGFR-1 (P < 0.001) and VEGFR-3 (P = 0.008) were prognostic indicators of MFS. PDGF-A (P = 0.012), PDGF-B (P = 0.015), PDGFR-α (P = 0.011), VEGF-A (P = 0.002) and VEGFR-1 (P = 0.036) were prognostic indicators of RFS.

**Table 2:** Angiogenic markers as predictors for disease-specific survival, metastasis and local recurrence in patients with resected soft-tissue sarcomas of the extremities or trunk (univariate analyses, log rank test, n = 115)

		**Disase-specific survival**		**Metastasis-free survival**		**Recurrence-free survival**	
**Marker expression**	**Patients (n)**	**5-Year survival (%)**	**P**	**5-Year survival (%)**	**P**	**5-Year survival (%)**	**P**
**PDGF-A**							
Low	54	60	0.035	74	0.007	79	0.012
High	58	52		51		55	
Missing	3						
**PDGF-B**							
Low	44	68	0.006	78	0.003	82	0.015
High	68	48		52		56	
Missing	3						
**PDGF-C**							
Low	31	71	0.032	68	0.214	68	0.564
High	80	50		61		65	
Missing	4						
**PDGF-D**							
Low	73	67	0.003	70	0.051	77	0.002
High	40	34		49		42	
Missing	2						
**PDGFR-α**							
Low	69	67	0.002	74	0.002	77	0.011
High	43	38		42		45	
Missing	3						
**PDGFR-β**							
Low	85	64	0.029	72	0.002	69	0.825
High	24	32		35		58	
Missing	6						
**VEGF-A**							
Low	60	65	0.001	75	0.001	77	0.002
High	51	43		48		51	
Missing	4						
**VEGF-C**							
Low	69	55	0.476	68	0.083	68	0.232
High	38	60		56		63	
Missing	8						
**VEGF-D**							
Low	84	57	0.131	67	0.081	70	0.177
High	29	50		51		55	
Missing	2						
**VEGFR-1**							
Low	67	63	0.002	77	<0.001	72	0.036
High	44	46		43		58	
Missing	4						
**VEGFR-2**							
Low	78	58	0.332	67	0.189	71	0.240
High	28	52		53		57	
Missing	9						
**VEGFR-3**							
Low	75	60	0.053	70	0.008	70	0.159
High	34	45		46		57	
Missing	6						
**FGF2**							
Low	75	61	0.033	66	0.214	70	0.648
High	35	49		56		67	
Missing	6						
**FGFR-1**							
Low	83	58	0.460	64	0.411	66	0.768
High	26	47		55		71	
Missing	6						

*Abbreviations*: *PDGF* Platelet-derived growth factor, *PDGFR* Platelet-derived growth factor receptor, *VEGF* Vascular endothelial growth factor, *VEGFR* Vascular endothelial growth factor receptor, *FGF* Fibroblast growth factor, *FGFR* Fibroblast growth factor receptor.

The impact of the clinicopathological variables on DSS, MFS and RFS in the VR group are summarized in Table 1. Age (P < 0.001), gender (P = 0.039), malignancy grade (P = 0.005) and resection margins (P = 0.021) were prognostic indicators of DSS. Gender (P = 0.022) was a prognostic indicator of MFS and tumor size (P = 0.006), malignancy grade (P = 0.046) and resection margins (P < 0.001) were prognostic indicators of RFS.

The impact of angiogenic markers on DSS, MFS and RFS in the VR group is summarized in Table 3. FGRF-1 (P = 0.023) was the only prognostic indicator for DSS and PDGF-C (P = 0.045) for RFS.

**Table 3:** Angiogenic markers as predictors for disease-specific survival, metastasis and local recurrence in patients with resected visceral & retroperitoneal soft-tissue sarcomas (univariate analyses, log rank test, n = 66)

		**Disase-specific survival**		**Metastasis-free survival**		**Recurrence-free survival**	
**Marker expression**	**Patients (n)**	**5-Year survival (%)**	**P**	**5-Year survival (%)**	**P**	**5-Year survival (%)**	**P**
**PDGF-A**							
Low	23	49	0.473	63	0.593	65	0.315
High	39	63		54		73	
Missing	4						
**PDGF-B**							
Low	14	54	0.604	82	0.088	61	0.291
High	48	59		51		73	
Missing	4						
**PDGF-C**							
Low	20	39	0.297	59	0.986	53	0.045
High	41	65		56		76	
Missing	5						
**PDGF-D**							
Low	48	52	0.078	52	0.197	70	0.343
High	15	80		73		59	
Missing	3						
**PDGFR-α**							
Low	41	56	0.672	61	0.527	71	0.761
High	21	61		51		66	
Missing	4						
**PDGFR-β**							
Low	58	57	0.360	54	0.532	70	0.766
High	4	75		75		75	
Missing	4						
**VEGF-A**							
Low	34	51	0.326	64	0.719	64	0.054
High	29	68		53		79	
Missing	3						
**VEGF-C**							
Low	34	66	0.402	69	0.071	62	0.051
High	29	50		45		84	
Missing	3						
**VEGF-D**							
Low	34	60	0.856	62	0.388	63	0.116
High	30	55		51		78	
Missing	2						
**VEGFR-1**							
Low	37	55	0.724	63	0.358	70	0.510
High	25	63		49		71	
Missing	3						
**VEGFR-2**							
Low	44	55	0.858	63	0.446	69	0.821
High	19	67		48		75	
Missing	3						
**VEGFR-3**							
Low	36	54	0.552	59	0.821	65	0.220
High	25	61		52		76	
Missing	5						
**FGF2**							
Low	39	56	0.805	51	0.214	74	0.748
High	20	65		67		66	
Missing	7						
**FGFR-1**							
Low	43	45	0.023	56	0.385	68	0.448
High	20	89		63		78	
Missing	3						

*Abbreviations*: *PDGF* Platelet-derived growth factor, *PDGFR* Platelet-derived growth factor receptor, *VEGF* Vascular endothelial growth factor, *VEGFR* Vascular endothelial growth factor receptor, *FGF* Fibroblast growth factor, *FGFR* Fibroblast growth factor receptor.

### Multivariate cox proportional hazards analysis

Table 4 presents multivariate analyses of clinicopathological and angiogenic marker variables with respect to DSS, MFS and RFS in the ET and VR groups, respectively.

**Table 4:** Multivariate analyses of clinopathological variables and angiogenic markers as prognostic values for disease-specific survival, metastasis and local recurrence in patients with resected soft-tissue sarcomas of the trunk or extremities (cox proportional hazards test)

	**Disase-specific survival**			**Metastasis-free survival**			**Recurrence-free survival**		
**Variable**	**HR**	**95% CI**	**P**	**HR**	**95% CI**	**P**	**HR**	**95% CI**	**P**
**Extremity & trunk**									
**Malignancy grade**									
1	1.000		<0.001*						
2	4.066	1.389-11.901	0.010						
3	6.025	2.058-17.634	0.001						
**Vascular invasion**									
Absent	1.000			1.000			1.000		
Present	2.141	1.188-3.859	0.011	5.284	2.418-11.544	<0.001	2.135	1.019-4.475	0.045
**Resection margins**									
Wide	1.000						1.000		
Non-wide	1.818	1.032-3.203	0.039				2.687	1.289-5.602	0.008
**PDGF-B**									
Low							*1.000*		
High							*2.099*	*0.937-4.706*	*0.072*
**PDGF-D**									
Low	1.000						*1.000*		
High	1.863	1.057-3.283	0.031				*1.844*	*0.931-3.653*	*0.079*
**VEGF-A**									
Low							1.000		
High							2.095	1.028-4.271	0.042
**VEGFR-1**									
Low				1.000					
High				2.106	1.038-4.272	0.039			
**Visceral & retroperitoneal**									
									
**Gender**									
Male				1.000					
Female				4.612	1.089-19.536	0.038			
**Malignancy grade**									
1	1.000		0.003*				1.000		0.061
2	4.812	1.823-12.705	0.002				2.069	0.630-6.794	0.231
3	5.646	1.790-17.804	0.003				5.665	1.330-24.123	0.019
**Resection margins**									
Wide	1.000						1.000		
Non-wide	2.712	1.222-6.018	0.014				11.996	3.128-46.005	<0.001
**PDGF-C**									
Low							*1.000*		
High							*0.413*	*0.157-1.089*	*0.074*
**FGFR-1**									
Low	1.000								
High	0.243	0.095-0.618	0.003						

*Abbreviations*: *HR* Hazard ratio, *CI* Confidence interval, *VEGF* Vascular endothelial growth factor, *VEGFR* Vascular endothelial growth factor receptor, *PDGFR* Platelet-derived growth factor receptor, *FGFR* Fibroblast growth factor receptor, *overall significance as prognostic factor.

In the ET group, high malignancy grade (P < 0.001), the presence of vascular invasion (P = 0.011), non-wide resection margins (P = 0.039) and high expression of PDGF-D (HR = 1.863, 95% CI = 1.057-3.283, P = 0.031) were significant independent prognostic indicators of DSS. Further, the presence of vascular invasion (P < 0.001) and high expression of VEGFR-1 (HR = 2.106, 95% CI = 1.038-4.272, P = 0.039) were significant independent prognostic factors of MFS, while the presence of vascular invasion (P = 0.045), non-wide resection margins (P = 0.008) and high expression of VEGF-A (HR 2.095, 95% CI 1.028-4.271, P = 0.042) were significant independent prognostic factors of RFS.

In the VR group, high malignancy grade (P = 0.003) and non-wide resection margins (P = 0.014) were significant independent adverse prognostic indicators of DSS whereas high FGFR-1 expression (HR = 0.243, 95% CI = 0.095-0.618, P = 0.003) was an independent positive prognostic indicator of DSS. Female gender (P = 0.038) was an independent negative prognostic indicator of MFS while non-wide resection margins (P < 0.001) was an independent negative prognostic indicator of RFS.

## Discussion and conclusions

In our univariate analyses high expression of most examined angiogenic markers were prognosticators of DSS and/or MFS and/or RFS in the ET group. Further, PDGF-D was an independent negative prognostic indicator of DSS, VEGFR-1 an independent negative prognostic indicator of MFS and VEGF-A an independent negative prognostic indicator of RFS. In contrast, only FGFR-1 was a prognosticator of DSS in both the univariate and multivariate analyses of the VR group. To our knowledge, this is the first comparison of the expression of angiogenic molecules in ET versus VR STSs.

Current knowledge of the importance of tumor localization (ET versusVR tumors) when it comes to the prognostic impact of angiogenic markers in STSs is limited. Yudoh et. al. investigated the level of VEGF-A in tissue from ET patients and found high levels to predict survival, local recurrence and metastasis [18]. We have previously reported on the expression of PDGFs, VEGFs and FGFs in a larger cohort of STS of mixed sites and histology and found high expression of VEGFR-3, PDGF-B and FGF2 to have independent negative prognostic impact on DSS [14-16]. When comparing the expression of angiogenic markers based on tumor location, it becomes apparent that these variables almost exclusively have prognostic impact in STS arising in the ET group (Tables 2, 3 and 4). This difference could to some extent be due to a smaller number of patients in the VR group, with a resulting increased risk of false negative results. However, near all angiogenic markers showed significant prognostic impact in the univariate analyses of the ET group, whereas only FGFR-1 showed prognostic impact in the VR group. Table 1 summarizes the clinopathological values in the ET and VR groups and it is apparent that the VR group contains a higher percentage of leiomysarcomas and liposarcomas. The different distribution of histologies between the ET and VR groups might suggest that angiogenic markers have higher impact in STSs arising in ET locations. Another explanation may be that ET tumors, even the slow growing ones, will produce symptoms when they reach a certain size due to limits created by connective and muscle tissue and blood and lymph vessels. VR tumors could in contrast grow to significant size before producing symptoms. This may explain our results as VR tumors in many cases only are found after the angiogenic switch have occurred, thus the impact of angiogenic markers have been negated in these tumors.

In the PDGF-axis, all markers were prognosticators of DSS, all but PDGF-C were prognosticators of MFS and all but PDGF-C and PDGFR-β were prognosticators of RFS in the ET group (Table 2), while none of the PDGFs were prognosticators in the VR group. Further, PDGF-D was found to be an independent negative prognostic factor for DSS in the ET group. In our previous study, PDGF-B was an independent prognosticator of DSS [15], and in this study PDGF-D is an independent prognosticator of DSS. PDGF-B binds all PDGFRs while PDGF-D binds PDGFR-αβ and-ββ [11]. Both PDGF-B and PDGF-D has been shown to exhibit similar and extensive angiogenic and transforming abilities [19,20]. Although our results cannot distinguish whether PDGF signalling drives tumor development through angiogenesis or other pathways, they strongly suggest PDGF signalling to be an important part of STS growth and progression.

In the VEGF-axis, VEGF-A, and VEGFR-1 were prognosticators of DSS, MFS and RFS in the ET group, while none of the VEGFs were prognosticators in the VR group (Table 2). Further, VEGFR-1 was an independent prognostic indicator of MFS and VEGF-A was an independent prognostic indicator of RFS in the ET group. VEGF-A signalling is the major angiogenic pathway, and high tumor expression and availability in serum has previously been associated with malignancy grade, metastasis, local recurrence and worse overall survival in STS patients [18,21-26]. VEGFR-1 is thought to modulate VEGF-A signalling through VEGFR-2, has anti-angiogenic properties in its soluble form, and has been linked to metastasis in experimental studies suggesting a feasible biological link for our finding in these STS patients [27,28]. This latter finding is quite interesting as antibodies and small-molecules targeting VEGFR-1 are being developed [29,30].

In the FGF-axis, FGF-2 was an unfavorable prognostic indicator of DSS in ET group. FGF2 is thought to drive cell-cycling, activate extracellular matrix remodelling and to rescue PDGF-B and VEGF-A driven angiogenesis in the presence of their respective inhibitors [13,31,32]. Surprisingly, FGFR-1 was an independent positive indicator of DSS in the VR group. To our knowledge these are new data, but these results have to be validated before a firm conclusion may be drawn due to the low number of patients.

This study enhances our current knowledge on angiogenic prognosticators in STSs, strongly indicates the involvement of the PDGF and VEGF pathways in ET STS development and adds to the growing body of evidence suggesting that STSs of different sites and histology should be analyzed independently in future studies. Further emphasis should also be put on validating VEGFR-1 as a predictor of MFS in ET STS patients, as these patients may benefit from adjuvant therapy targeting VEGFR-1.

## Competing interests

The authors declare that they have no competing interests.

## Authors’ contributions

All authors participated in designing the study, interpreting the results and in the writing of the paper. TK, AV, SWS and ES collected the clinical and demographic data. TK, SWS and AV scored the TMAs. TK and TD conducted the statistical analyses. TK drafted the manuscript. All authors read and approved the final manuscript.

## Pre-publication history

The pre-publication history for this paper can be accessed here:

http://www.biomedcentral.com/1472-6890/14/5/prepub
